# Polyphenolic Characterization and Antioxidant Activity of *Malus domestica* and *Prunus domestica* Cultivars from Costa Rica

**DOI:** 10.3390/foods7020015

**Published:** 2018-01-30

**Authors:** Mirtha Navarro, Ileana Moreira, Elizabeth Arnaez, Silvia Quesada, Gabriela Azofeifa, Felipe Vargas, Diego Alvarado, Pei Chen

**Affiliations:** 1 Department of Chemistry, University of Costa Rica (UCR), Rodrigo Facio Campus, San Pedro Montes Oca, San Jose 2060, Costa Rica; luis.vargashuertas@ucr.ac.cr; 2 Department of Biology, Technological University of Costa Rica (TEC), Cartago 7050, Costa Rica; imoreira@itcr.ac.cr (I.M.); earnaez@itcr.ac.cr (E.A.); 3 Department of Biochemistry, School of Medicine, University of Costa Rica (UCR), Rodrigo Facio Campus, San Pedro Montes Oca, San Jose 2060, Costa Rica; silvia.quesada@ucr.ac.cr (S.Q.); gabriela.azofeifacordero@ucr.ac.cr (G.A.); 4 Department of Biology, University of Costa Rica (UCR), Rodrigo Facio Campus, San Pedro Montes Oca, San Jose 2060, Costa Rica; luis.alvaradocorella@ucr.ac.cr; 5 Food Composition and Methods Development Laboratory, Beltsville Human Nutrition Research Center, Agricultural Research Service, U.S. Department of Agriculture, MD 20705, USA; pei.chen@ars.usda.gov

**Keywords:** *Malus domestica*, *Prunus domestica*, apple, plum, UPLC, ESI-MS, proanthocyanidins, flavonoids, mass spectrometry, antioxidant

## Abstract

The phenolic composition of skin and flesh from *Malus domestica* apples (Anna cultivar) and *Prunus domestica* plums (satsuma cultivar) commercial cultivars in Costa Rica, was studied using Ultra Performance Liquid Chromatography coupled with High Resolution Mass Spectrometry (UPLC-DAD-ESI-MS) on enriched-phenolic extracts, with particular emphasis in proanthocyanidin and flavonoids characterization. A total of 52 compounds were identified, including 21 proanthocyanidins ([(+)-catechin and (−)-epicatechin]) flavan-3-ols monomers, five procyanidin B-type dimers and two procyanidin A-type dimers, five procyanidin B-type trimers and two procyanidin A-type trimers, as well as one procyanidin B-type tetramer, two procyanidin B-type pentamers, and two flavan-3-ol gallates); 15 flavonoids (kaempferol, quercetin and naringenin derivatives); nine phenolic acids (protochatechuic, caffeoylquinic, and hydroxycinnamic acid derivatives); five hydroxychalcones (phloretin and 3-hydroxyphloretin derivatives); and two isoprenoid glycosides (vomifoliol derivatives). These findings constitute the first report of such a high number and diversity of compounds in skins of one single plum cultivar and of the presence of proanthocyanidin pentamers in apple skins. Also, it is the first time that such a large number of glycosylated flavonoids and proanthocyanidins are reported in skins and flesh of a single plum cultivar. In addition, total phenolic content (TPC) was measured with high values observed for all samples, especially for fruits skins with a TPC of 619.6 and 640.3 mg gallic acid equivalents/g extract respectively for apple and plum. Antioxidant potential using 2,2-diphenyl-1-picrylhidrazyl (DPPH) and oxygen radical absorbance capacity (ORAC) methods were evaluated, with results showing also high values for all samples, especially again for fruit skins with IC_50_ of 4.54 and 5.19 µg/mL (DPPH) and 16.8 and 14.6 mmol TE/g (ORAC) respectively for apple and plum, indicating the potential value of these extracts. Significant negative correlation was found for both apple and plum samples between TPC and DPPH antioxidant values, especially for plum fruits (*R* = −0.981, *p* < 0.05) as well as significant positive correlation between TPC and ORAC, also especially for plum fruits (*R* = 0.993, *p* < 0.05) and between both, DPPH and ORAC antioxidant methods (*R* = 0.994, *p* < 0.05).

## 1. Introduction

Polyphenols effects on health are based on results obtained from bioactivity studies, which in turn, has increased the interest in the consumption of foods and beverages rich in polyphenols as well as the importance of related scientific research. Such bioactive effects include antioxidant properties, prevention of oxidative stress associated diseases like cardiovascular, neurodegenerative diseases and cancer [1] and their role in long-term health protection by reducing the risk of chronic and degenerative diseases [2].

Several studies have linked vegetable consumption, specially fruits with a reduced risk for cancer and cardiovascular disease, thus characterization of polyphenols is essential to increase the knowledge on fruits contents and their related beneficial effects. *Malus domestica* (apple) and *Prunus domestica* (plum) are trees from the Rosaceae family, both native from southern Europe and western Asia that were introduced in Costa Rica as an initiative of local producers to diversify their crops, constitute fruits of high consumption in the country.

Studies on *M. domestica* have found these fruits to have a potent antioxidant activity and to inhibit the growth of cancer cells in vitro [3,4]. Similar properties have been described for plum [5,6] and some of these effects were attributed at least partially to its polyphenolic contents.

Previous studies on polyphenols of *M. domestica* have shown mainly flavonoids, phenolic acids, and chalcones, while proanthocyanidins included only catechin and epicatechin monomers and procyanidin dimers [7,8,9] and other reporting also trimers mainly in skin [10,11]. In the case of *P. domestica*, studies have focused mostly in a specific type of compounds, such as caffeolylquinic acid derivatives [12,13], flavonoids such as quercetin derivatives [14], and both type of compounds [15]. Few reports have studied also extensively proanthocyanidins, findings indicating mainly monomers and procyanidin dimers [16,17].

However other findings [18] would suggest proanthocyanidins with higher polymerization degree, without having reported their particular characterization. Despite the increasing number of studies on phenolics, the characterization of proanthocyanidins remains a complex task because of the need for high-end techniques such as high-resolution mass spectroscopy (HRMS). Proanthocyanidins, which are condensed flavan-3-ols, constitute an important group of polyphenols because of their bioactivities, for instance, mainly to their antioxidant capacity, which in turn is linked, among others, with their ant-inflammatory and anti-cancer activities [19].

Antioxidant properties have been reported to increase with proanthocyanidins degree of polymerization and the presence of specific structures such as procyanidin tetramers and pentamers has been found to enhance such properties [20,21], thus further knowledge on these phenolic structures characterization in apple and plum would contribute to a better understanding of their implications in the fruits quality as a source of dietary compounds with potential biological properties. Therefore, the objective of the present work was to obtain enriched polyphenolic extracts of fruits from *M. domestica* and *P. domestica* commercial cultivars in Costa Rica, and to characterize them through Ultra Performance Liquid Chromatography coupled with High Resolution Mass Spectrometry (UPLC-DAD-ESI-MS), with particular emphasis in flavonoids and proanthocyanidins. Evaluation of the total polyphenolic contents and antioxidant activity using 2,2-diphenyl-1-picrylhidrazyl (DPPH) and oxygen radical absorbance capacity (ORAC) methods, was also carried out in the different extracts.

## 2. Results and Discussion

### 2.1. Phenolic Yield and Total Phenolic Contents

The extraction process described in the Materials and Methods section, allowed to obtain phenolic enriched extracts, as summarized in Table 1. *P. domestica (satsuma cultivar)* skin presented the highest yield (2.74%) whereas *M. domestica (Anna cultivar)* flesh showed the lowest value (0.51%). In both fruits, skin extract yields were higher than flesh extracts. The total phenolic contents (TPC) shown also in Table 1, resulted in high values for all samples with both skins exhibiting slightly higher results, ranging from 619.6–640.3 gallic acid equivalents (GAE)/g dry extract.

Results from literature show variability among reports from other apple cultivars with total phenolic contents (TPC) values ranging between 0.3–25.9 mg GAE/g DW for skin and 1.6–14.8 mg GAE/g DW for flesh [22,23,24,25]. Our results for Anna cultivar skin and flesh 7.4 and 3 mg GAE/g DW respectively (values calculated using TPC and extract yields from Table 1) are within that range. A similar situation occurs in the case of plum, with determination of total phenolic contents in the literature revealing variability, with values ranging between 18.4–495 mg GAE/100 g fresh weight (FW) [14,26,27], whereas our findings of 109–179 mg GAE/100 g FW (values calculated using TPC, extract and lyophilization yields from Table 1) are in agreement with the published results.

### 2.2. Profile by UPLC-DAD-ESI-TQ-MS Analysis

**Figure 1:**
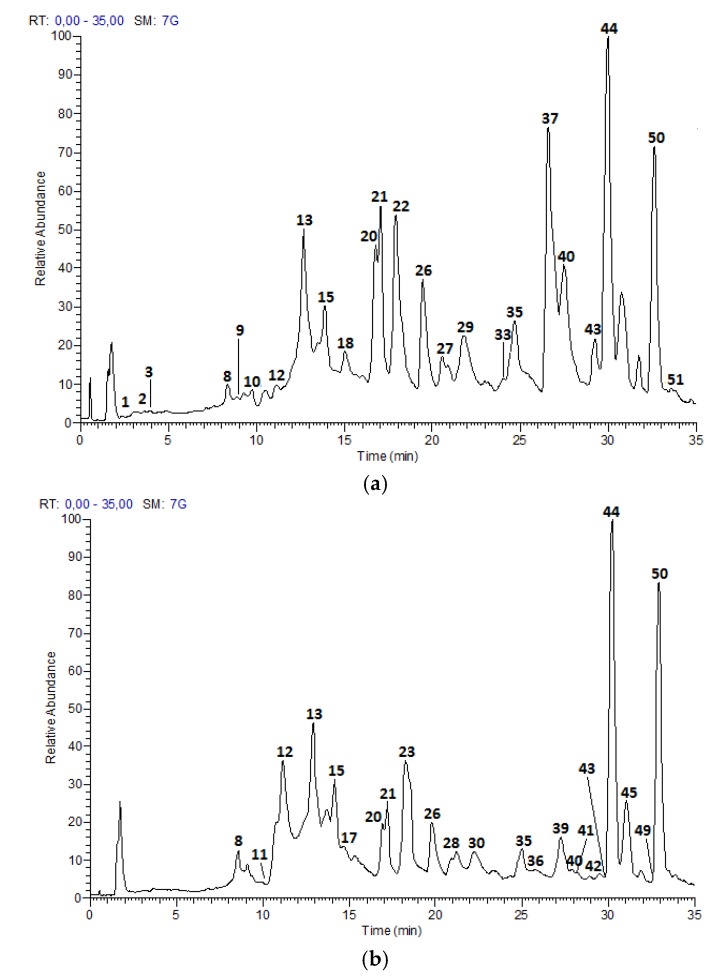
HPLC Chromatograms of *M. domestica* extracts: (**a**) Anna skins (**b**) Anna flesh, in a Hypersil Gold AQ RP-C18 column (200 mm × 2.1 mm × 1.9 µm) using a LTQ Orbitrap XL Mass spectrometer (Thermo Scientific™, Walthman, MA, USA) in a mass range from 100 to 2000 amu.

**Figure 2:**
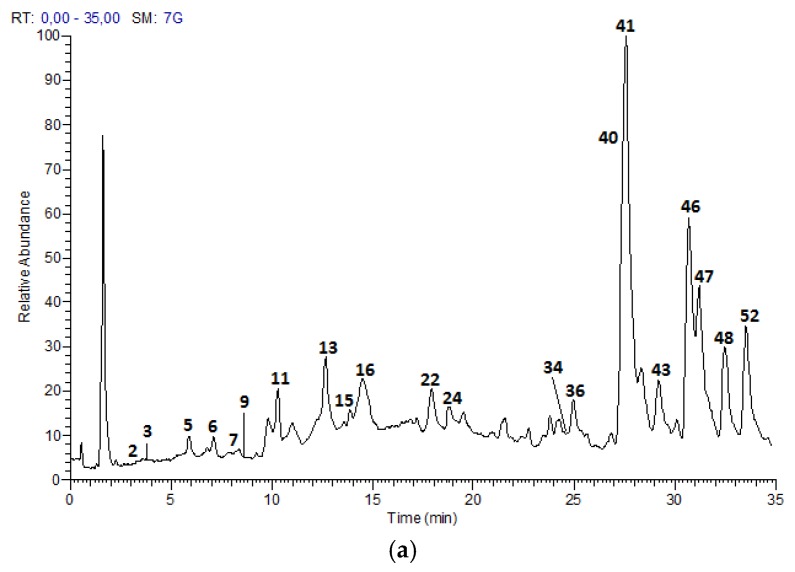

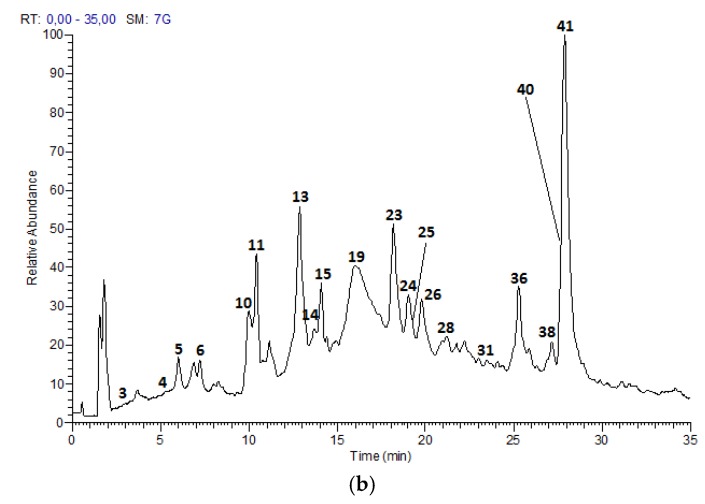
HPLC Chromatograms of *P. domestica* extracts: (**a**) Satsuma skin (**b**) Satsuma flesh in a Hypersil Gold AQ RP-C18 column (200 mm × 2.1 mm × 1.9 µm) using a LTQ Orbitrap XL Mass spectrometer (Thermo Scientific™, Walthman, MA, USA) in a mass range from 100 to 2000 amu.

The UPLC-DAD-ESI-MS/MS analysis described in the Materials and Methods section, allowed to identify 52 compounds, including 21 proanthocyanidins and flavan-3-ol monomers, 15 glycosylated flavonols and nine acids and derivatives in Costa Rican apple (Anna cultivar) and plum (Satsuma cultivar) only commercial cultivars; as well as five hydroxy chalcones and two glycosylated isoprenoids characteristic of apple fruits. Figure 1 and Figure 2 show the chromatograms of the samples and Table 2 summarizes the analysis results for the 52 compounds.

The first common group of compounds, proanthocyanidins, corresponded to oligomers of flavan-3-ols catechin and epicatechin. The monomeric units of these proanthocyanidins are linked through a C4-C8 or C4-C6 bond (B-type), which coexist with an additional C2-O-C7 linkage (A-type) [28]. Peaks 9 (Rt = 8.87 min) and 15 (Rt = 13.97 min) showed a [M − H]^−^ at *m*/*z* 289.0710 (C_15_H_14_O_6_) that correspond to monomers catechin or epicatechin. The main MS^2^ fragments at *m*/*z* 245 and 205, occur through the loss of C_2_H_4_O and C_4_H_4_O_2_ due to retro-Diels-Alder fission (RDA) of ring A [29].

**Figure 3:**
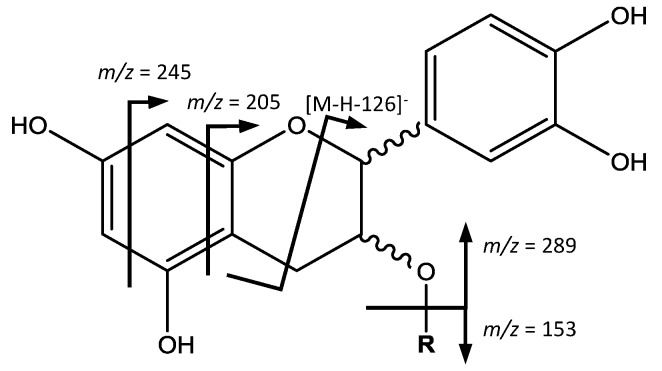
Flavan-3-ols monomers and gallates structure and main fragments.

On the other hand, peaks 3 (Rt = 3.96 min), 4 (Rt = 5.40 min), whose [M − H]^−^ is at *m*/*z* 441.0819 (C_22_H_18_O_10_) correspond to (epi)catechin-3-*O*-gallate (Figure 3). The main fragment at *m*/*z* 315 [M – H − 126]^−^ is due to the elimination of a phloroglucinol, and fragments at *m*/*z* 289 and 153 are both residuals from the cleavage of the ester group [30].

**Figure 4:**
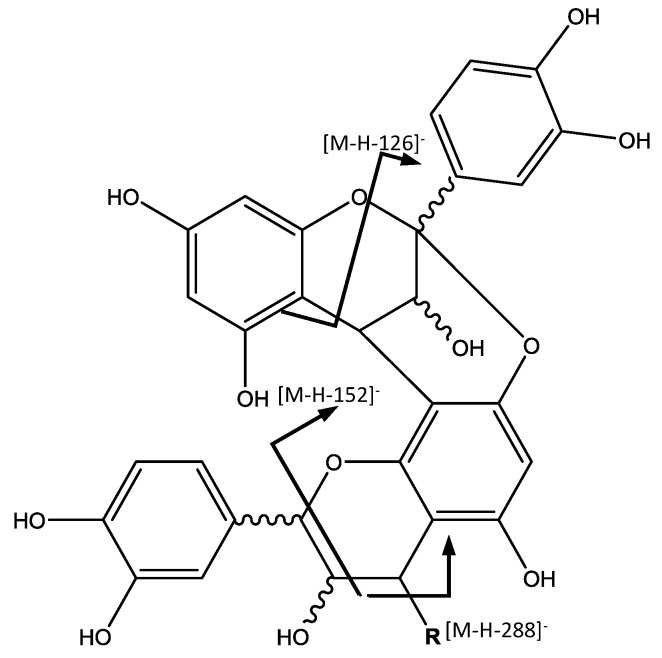
Proanthocyanidin A-type structure and main fragments.

Peak 36 (Rt = 25.19 min) shows a [M − H]^−^ at *m*/*z* 575.1185 (C_30_H_24_O_12_) and main MS^2^ ion at *m*/*z* 449, which indicate the presence of a procyanidin A-type dimer. The base ion at *m*/*z* 449 [M – H − 126]^−^, corresponds to the elimination of a phloroglucinol molecule from this A-type dimer [31]. Peaks 24 (Rt = 18.98 min), 25 (Rt = 19.37 min) and 31 (Rt = 23.54 min) show a [M − H]^−^ at *m*/*z* 863.1798 (C_45_H_36_O_18_), revealing the presence of a procyanidin trimer with A-type interflavan linkage (Figure 4). In the MS^2^ spectrum, fragment ions at *m*/*z* 711 [M – H − 152]^−^ and 575 [M – H − 288]^−^ observed, result from the RDA fission and quinone-methide (QM) cleavage, respectively [32].

**Figure 5:**
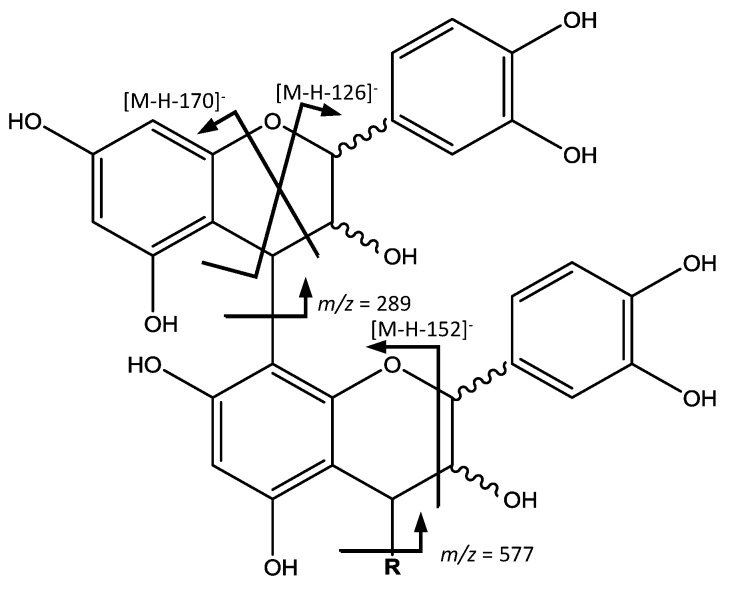
Proanthocyanidin B-type structure and main fragments.

Peaks 1 (Rt = 2.69 min), 8 (Rt = 8.44 min), 13 (Rt = 12.75 min), 38 (Rt = 27.21 min) and 42 (Rt = 28.62 min) show [M − H]^−^ at *m*/*z* 577.1344 (C_30_H_26_O_12_), corresponding to procyanidins with B-type linkage (Figure 5), 2 amu (atomic mass units) higher than that of the A-type procyanidin, and major ions containing the structural information at *m*/*z* 559, 451, 425, 407 and 289. The ion at *m*/*z* 559 [M – H − 18]^−^ originates from water loss. The ion at *m*/*z* 451 [M – 126 − H]^−^ results from the elimination of the phloroglucinal as in A-type dimers. The fragment ions at *m*/*z* 425 [M – H − 152]^−^ and 407 [M – H − 170]^−^ come from RDA, while the ion at *m*/*z* 289 originates from QM resulting in the ion of the monomer [31].

On the other hand (Figure 5), peaks 14 (Rt = 13.20 min), 22 (Rt = 17.88 min), 23 (Rt = 18.16 min), 28 (Rt = 21.23 min) and 30 (Rt = 22.21 min) with ([M − H]^−^) at *m*/*z* 865.1956 (C_45_H_38_O_18_) were tentatively identified to be procyanidin B-type trimers. Their fragmentation behaviors seem to be similar to that of dimers, with ion fragments at *m*/*z* 695 [M – H − 170]^−^, 713 [M – H − 152]^−^ and 739 [M – H − 126]^−^. The QM cleavage of the interflavan bond mainly produced the ions at *m*/*z* 289 and 577, indicating the cleavage happens in upper interflavan bond [31].

In a similar way (Figure 5), peak 26 (Rt = 19.70 min), [M − H]^−^ at *m*/*z* 1153.2603 (C_60_H_50_O_24_) was identified as a procyanidin B-type tetramer, with fragments at *m*/*z* 1027 [M – H − 126]^−^, 1001 [M – H − 152]^−^, 983 [M – H − 170]^−^, 865 and 577. Also, peaks 27 (Rt = 20.54 min) and 29 (Rt = 21.83 min), with [M − H]^−^ at *m*/*z* 1441.3229 (C_75_H_62_O_30_), were identified as two procyanidin B-type pentamers with a characteristic fragment at *m*/*z* 1315 [M – H − 126]^−^, and also those derived from QM cleavage at *m*/*z* 1151, 865, 577 and 289 [33].

**Figure 6:**
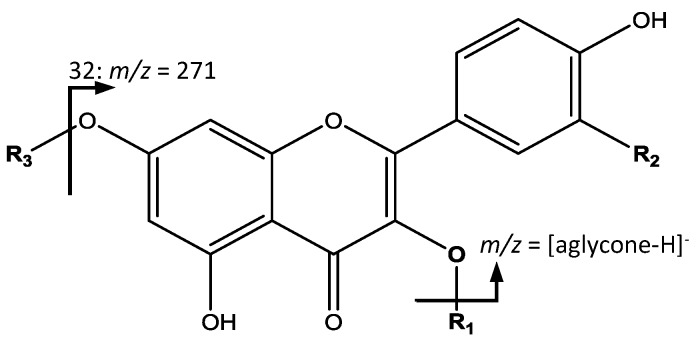
Flavonol glycosides structure and main fragments.

The second group of common compounds, glycosylated flavonol derivatives were elucidated based in the fragmentation pattern from the aglycone due to the loss of glycosides (Figure 6). For instance, peaks 18 (Rt = 14.97 min) and 19 (Rt = 15.90 min) had [M − H]^−^ at *m*/*z* 447.0922 (C_21_H_20_O_11_) were identified as kaempferol-hexoside isomers with a main fragment at *m*/*z* 285 corresponding to kaempferol [34]. Peak 32 (Rt = 23.97 min) with [M − H]^−^ at *m*/*z* 433.1131 (C_21_H_22_O_10_) showed its main fragment at *m*/*z* 271, corresponding to naringenin-hexoside [35].

All the remaining flavonol derivatives presented their main fragment at *m*/*z* 301, which corresponds to the quercetin aglycone. They differ in the bonded glycoside with some variations among them. For instance, peaks 33 (Rt = 24.12 min) and 34 (Rt = 24.72 min) had a [M − H]^−^ at *m*/*z* 595.1284 (C_26_H_28_O_16_), were assigned to quercetin-pentosylhexoside isomers. Peaks 37 (Rt = 26.57 min) and 41 (Rt = 27.79 min), with [M − H]^−^ at *m*/*z* 463.0875 (C_21_H_20_O_12_) correspond to quercetin-hexoside isomers. Peak 40 (Rt = 27.60 min), with [M − H]^−^ at *m*/*z* 609.1440 (C_27_H_30_O_16_) was identified as quercetin-rutinoside. Peaks 43 (Rt = 29.28 min) and 46 (Rt = 30.69 min) had [M − H]^−^ at *m*/*z* 433.0769 (C_20_H_18_O_11_), coincident with isomers of quercetin-pentoside [36].

Peak 47 (Rt = 31.21 min) had [M − H]^−^ at *m*/*z* 565.1184 (C_25_H_26_O_15_), were identified as quercetin-pentosyl-pentoside. Peaks 48 (Rt = 32.46 min), 49 (Rt = 32.57 min) and 51 (Rt = 33.27 min) with [M − H]^−^ at *m*/*z* 447.0922 (C_21_H_20_O_11_) were assigned as quercetin-deoxyhexoside isomers. Finally, peak 52 (Rt = 33.51 min) with [M − H]^−^ at *m*/*z* 505.0975 (C_23_H_22_O_13_), was identified as quercetin-acetylhexoside [16].

**Figure 7:**
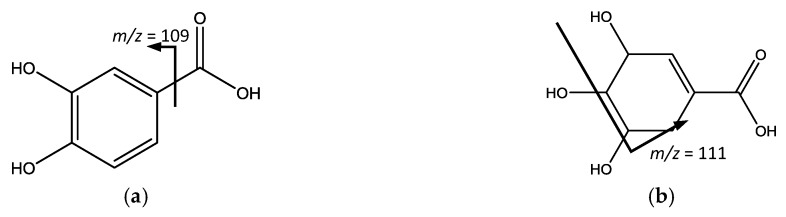
(**a**) Protochatechuic acid and (**b**) Shikimic acid structure and main fragments.

Among the third group of common compounds, acids and derivatives, two small acids correspond (Figure 7) to peak 2 (Rt = 3.36 min) with [M − H]^−^ at *m*/*z* 153.0191 (C_7_H_6_O_4_) and a main fragment at *m*/*z* 109 [M – H − 44]^−^ due to the loss of CO_2_ from a carboxylic acid [37] identified as protocatechuic acid, and peak 16 (Rt = 14.44 min), with [M − H]^−^ at *m*/*z* 173.0454 (C_7_H_10_O_5_) and main fragments at *m*/*z* 111 generated from RDA fission, and 93 from subsequent loss of water assigned to shikimic acid [38].

**Figure 8:**
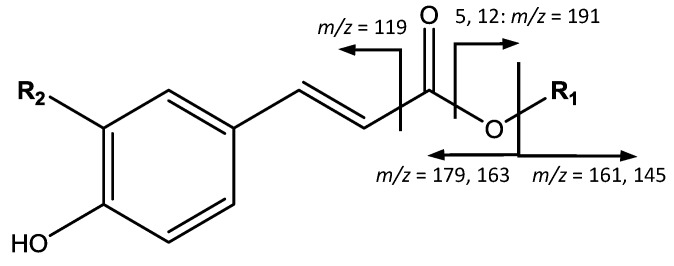
*p*-coumaric acid derivatives structure and main fragments.

On the other hand, a series of *p*-coumaric acid derivatives was identified, as shown in Figure 8. For instance, peaks 5 (Rt = 5.95 min) and 12 (Rt = 11.10 min), with [M − H]^−^ at *m*/*z* 353.0869 (C_16_H_18_O_9_), are identified as caffeoylquinic acid isomers, with main fragments at *m*/*z* 191 [quinic acid−H]^−^, 179 [caffeic acid−H]^−^, and 145 due to the loss of CO_2_ from the quinic acid ion. Peak 6 (Rt = 7.23 min) shows [M − H]^−^ at *m*/*z* 341.0872 (C_15_H_18_O_9_), with main fragments at *m*/*z* 179 [caffeic acid − H]^−^ and 161 [M – H − 179]^−^, corresponding to caffeoyl-hexoside. Peak 7 (Rt = 8.30 min) with [M − H]^−^ at *m*/*z* 163.0398 (C_9_H_6_O_3_), is identified as coumaric acid due to the fragment at 119 [M – H − CO_2_]^−^. Peaks 10 (Rt = 9.94 min) and 11 (Rt = 10.27 min), with [M − H]^−^ at *m*/*z* 325.0921 (C_15_H_18_O_8_) are assigned to coumaroyl-hexoside isomers, due to fragments at *m*/*z* 163 [coumaric acid−H]^−^ and 145 [coumaric acid−H−H_2_O]^−^. Another cinnamic acid derivative was found in peak 17 (Rt = 14.49 min) with [M − H]^−^ at *m*/*z* 337.0927 (C_16_H_18_O_8_), and a main fragment at *m*/*z* 173 due to the loss of water of the quinic acid ion, thus corresponding to *p*-coumaroylquinic acid [16].

**Figure 9:**
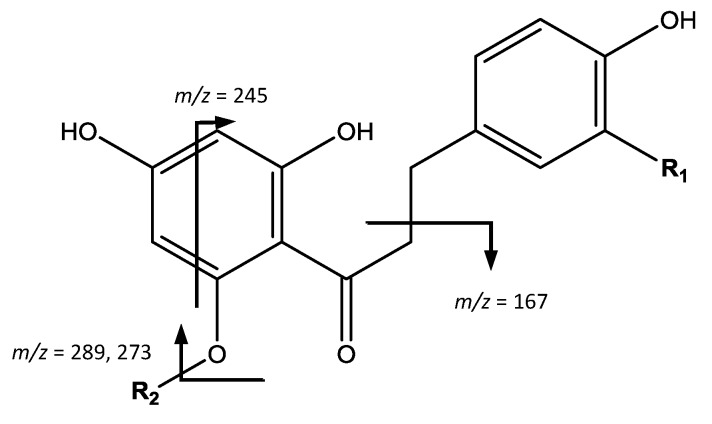
Chalcones structure and main fragments.

The fourth group of compounds, chalcones, shown in Figure 9, were found only in apples. For instance, peak 35 (Rt = 24.87 min) shows [M − H]^−^ at *m*/*z* 583.1660 (C_26_H_32_O_15_) and the main fragment at *m*/*z* 289, which correspond to 3-hydroxyphloretin aglycone, allowing to identify the compound as 3-hydroxyphloretin-pentosylhexoside [11]. Peak 39 (Rt = 27.29 min) shows [M − H]^−^ at *m*/*z* 289.0716 (C_15_H_14_O_6_), the same mass and molecular formula of flavan-3-ols, but differs in the fragmentation that occurs at *m*/*z* 271 [M – H − H_2_O]^−^ due to the loss of water, 245 coming from RDA, and 167 due to α-cleavage of the carbonyl group [39] therefore being assigned as 3-hydroxyphloretin.

On the other hand, peaks 44 (Rt = 30.11 min) and 45 (Rt = 31.04 min) with [M − H]^−^ at *m*/*z* 567.1704 (C_26_H_32_O_14_) were identified as phloretin-pentosylhexoside isomers, due to the main fragment at *m*/*z* 273 which corresponds to the phloretin ion generated from loss of glycosides. Finally, peak 50 (Rt = 32.78 min), [M − H]^−^ at *m*/*z* 273.0767 (C_15_H_14_O_5_) is assigned as phloretin, with a main fragment at *m*/*z* 167 due to α-cleavage of the carbonyl group [11,39].

**Figure 10:**
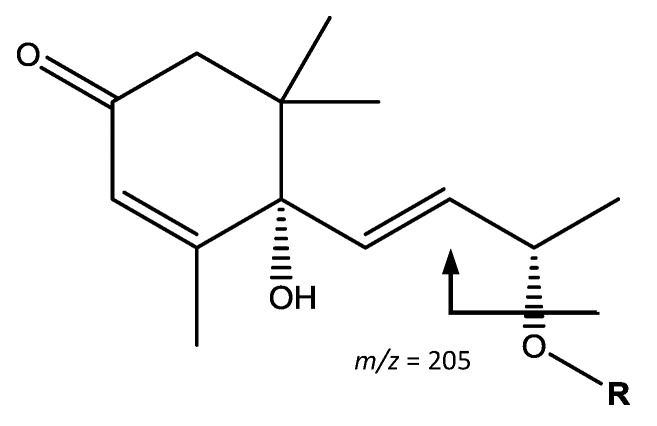
Vomifoliol-pentosylhexoside structure and main fragments.

The fifth group of compounds, glycosylated isoprenoid derivatives, shown in Figure 10, was found only in apple. Peaks 20 (Rt = 16.80 min) and 21 (Rt = 17.10 min) showed [M − H]^−^ at *m*/*z* 517.2280 (C_24_H_38_O_12_). Their main fragments at *m*/*z* 385 [M – H − 132]^−^ and 205 [M – H − 312]^−^ correspond to the loss of a pentoside and a pentosylhexoside respectively. The resulting ion is coincident with vomifoliol, allowing the peaks to be assigned to vomifoliol-pentosylhexoside isomers [40].

Polyphenol profiling reveals great similarities between skins and flesh, with a high number of compounds and high diversity for both fruits. When comparing the reports for apple cultivars in the literature, our results for Anna cultivar from Costa Rica show greater number and diversity of polyphenols than the findings on sixteen cultivars from Norway, Italy, Canada and United States [7,8,9,10], and similar to only two cultivars, namely Golden Delicious and Braeburn from Slovenia [11]. Further, findings on proanthocyanidins indicate better results both in total occurrence and in greater polymerization degree, for instance procyanidin tetramers and pentamers in the skins of Costa Rican Anna apple cultivar.

In the case of plums, when comparing the reports of twenty-four cultivars from United States, Germany and Portugal [15,16,17,18], greater number and diversity of polyphenols is observed in the skins of Satsuma cultivar from Costa Rica. Likewise, results for Satsuma flesh are also superior to twenty-three of the cultivars, being similar to President cultivar from Germany. Of special interest is the presence of a greater number of procyanidins oligomers, such as procyanidin trimers and one tetramer as well as of glycosylated flavonols, showing different quercetin derivatives not reported in previous characterizations of plum skins.

It is important to highlight the characterization of proanthocyanidin oligomers present in both fruits, which is of special interest due to procyanidins antioxidant results having been reported to increase with higher degree of polymerization, for instance trimers, tetramers and pentamers showing better results than dimers or flavan-3-ol monomers [20,21]. The detailed characterization of these fruit cultivars provides an important contribution expanding the knowledge for their exploitation as available sources of such diversity of polyphenols, which in turn can be of interest for further research due to their potential biological activities.

### 2.3. Antioxidant Activity

The DPPH and ORAC values obtained are summarized in Table 3. All samples show high antioxidant values and in both antioxidant tests, skins have better results than flesh, with Anna skin presenting the highest value with IC_50_ = 4.54 μg/mL for DPPH and 16.78 mmol Trolox equivalents/g for ORAC. Regarding antioxidant values from the literature, while no comparable results are available for plum fruits, evaluation of methanolic and aqueous extracts from Kahsmir cultivar apple skins show DPPH IC_50_ values of 55.54 µg/mL and 41.41 µg/mL respectively [41], thus Anna cultivar extracts showing better results. DPPH values expressed as mmol TE/g extract were obtained as described in the experimental section (in respect to Trolox IC_50_ = 5.62 μg/mL) and allow to compare our results with those reported in the literature for different extracts, for instance our values fit in the range between enju and grape seed extracts (0.76–1.35 mmol TE/g extract) used as antioxidant food additives [42,43].

On the other hand, for ORAC, a study of ethanolic extracts of Pelingo cultivar apples [10], reported values of 44.07 μmol Trolox eq./g DW for skin and 23.19 μmol Trolox eq./g DW for flesh, while findings for aqueous extracts showed values of 42.97 μmol Trolox eq./g DW and 31.99 μmol Trolox eq./g DW for skin and flesh respectively, therefore ORAC of extracts from Anna cultivar apples are superior for both skin and flesh since our findings indicate values of 57.33 μmol Trolox eq./g DW for flesh and 202.03 μmol Trolox eq./g DW (values calculated using ORAC from Table 3 and extract yields from Table 1).

The difference in antioxidant values among extracts could be attributed to the differences in their phenolic content and distribution. Thus, in order to investigate if the total phenolic contents (TPC, Table 1) contributes to the antioxidant activity, a correlation analysis was carried out between these TPC values with DPPH and ORAC results. Significant positive correlation (*p* < 0.05) was found for both apple and plum samples between TPC values and ORAC with *R* = 0.827 and R = 0.993 respectively, as well as significant negative correlation (*p* < 0.05) between TPC results and DPPH with *R* = −0.833 and *R* = −0.981 respectively. Therefore, our results are in agreement with previous studies reporting correlation between total polyphenolic contents and ORAC antioxidant activity [44]. Finally, our findings indicate positive correlation (*p* < 0.05) between both DPPH and ORAC antioxidant values (*R* = 0.994), thus in agreement with previous studies [43].

## 3. Materials and Methods

### 3.1. Materials, Reagents and Solvents

*Malus domestica* and *Prunus domestica* fruits were acquired in ripe state from FRUTALCOOP, a local producer cooperative located in Los Santos in Costa Rica. Cultivars were confirmed with the support of the Costa Rican National Herbarium and vouchers are deposited there. Reagents, such as fluorescein, 2,2-azobis(2-amidinopropane) dihydrochloride (AAPH), 2,2-diphenyl-1-picrylhidrazyl (DPPH), Trolox, gallic acid, and Amberlite XAD-7 resin were provided by Sigma-Aldrich (St. Louis, MO, USA), while solvents such as acetone, chloroform and methanol were purchased from Baker (Center Valley, PA, USA).

### 3.2. Phenolic Extracts from M. domestica and P. domestica Fruits

*M. domestica* and *P. domestica* fruits were rinsed in water, peeled, and both, skin and flesh material were freeze-dried in a Free Zone −105 °C, 4.5 L, Cascade Benchtop Freeze Dry System (Labconco, Kansas, MO, USA), and the freeze-dried material was preserved at −20 °C until extraction. Freeze-dried samples were extracted in a Dionex™ ASE™ 150 Accelerated Solvent Extractor (Thermo Scientific™, Walthman, MA, USA) using acetone:water (70:30) as solvent in a 34 mL cell, at 40 °C. Next, the extract was evaporated under vacuum to eliminate the acetone and the aqueous phase was washed with ethyl acetate and chloroform to remove less-polar compounds. Afterwards, the aqueous extract was evaporated under vacuum to eliminate organic solvent residues and was eluted (2 mL/min) in Amberlite XAD7 column (150 mm × 20 mm), starting with 300 mL of water to remove sugars, and then with 200 mL each of methanol:water (80:20) and pure methanol to obtain the polyphenols. Finally, the enriched extract was obtained after evaporating to dryness using a Buchi™ 215 (Flawil, Switzerland) rotavapor.

### 3.3. Total Phenolic Content

The polyphenolic content was determined by a modification of the Folin-Ciocalteu (FC) method [45], whose reagent is composed of a mixture of phosphotungstic and phosphomolybdic acids. Each sample was dissolved in MeOH (0.1% HCl) and combined with 0.5 mL of FC reagent. Afterwards 10 mL of Na_2_CO_3_ (7.5%) were added and the volume was completed to 25 mL with water. Blanks were prepared in a similar way but using 0.5 mL of MeOH (0.1% HCl) instead of sample. The mixture was let standing in the dark for 1 h and then absorbance was measured at 750 nm. Values obtained were extrapolated in a gallic acid calibration curve. Total phenolic content was expressed as mg gallic acid equivalents (GAE)/g sample. Analyses were performed in triplicate.

### 3.4. UPLC-DAD-ESI-TQ-MS Analysis

The UPLC-MS system used to analyze the composition of *M. domestica* and *P. domestica* fruit extracts consisted of an LTQ Orbitrap XL mass spectrometer with an Accela 1250 binary Pump, a PAL HTC Accela TMO autosampler, a PDA detector (Thermo Fisher Scientific, San Jose, CA, USA), and a G1316A column compartment (Agilent, Palo Alto, CA, USA). Separation was carried out on a Hypersil Gold AQ RP-C18 UHPLC column (200 mm × 2.1 mm i.d., 1.9 µm, Thermo Fisher Scientific) with an UltraShield pre-column filter (Analytical Scientific Instruments, Richmond, CA, USA) at a flow rate of 0.3 mL/min. Mobile phases A and B consist of a combination of 0.1% formic acid in water, *v*/*v* and 0.1% formic acid in acetonitrile, *v*/*v*, respectively. The linear gradient is from 4% to 20% B (*v*/*v*) at 20 min, to 35% B at 30 min and to 100% B at 31 min, and held at 100% B to 35 min. The UV/Vis spectra were acquired from 200–700 nm.

Negative electrospray ionization mode was used and the conditions were set as follows: sheath gas, 70 (arbitrary units); aux and sweep gas, 15 (arbitrary units); spray voltage, 4.8 kV; capillary temperature, 300 °C; capillary voltage, 15 V; tube lens, 70 V. The mass range was from 100 to 2000 amu with a resolution of 30,000, FTMS AGC target at 2 × 10^5^, FT- MS/MS AGC target at 1 × 10^5^, isolation width of 1.5 *amu*, and max ion injection time of 500 ms. The most intense ion was selected for the data-dependent scan to offer their MS^2^ to MS^5^ product ions, respectively, with a normalization collision energy at 35%.

### 3.5. DPPH Radical-Scavenging Activity

DPPH evaluation was performed as previously reported [46] and was expressed as IC_50_ (µg/mL), which is the amount of sample required to reach the 50% radical-scavenging activity, and also as mmol of Trolox equivalents (TE)/g extract. Briefly, a solution of 2,2-diphenyl-1-picrylhidrazyl (DPPH) (0.25 mM) was prepared using methanol as solvent. Next, 0.5 mL of this solution were mixed with 1 mL of extract or Trolox at different concentrations, and incubated at 25 °C in the dark for 30 min. DPPH absorbance was measured at 517 nm. Blanks were prepared for each concentration. The percentage of the radical-scavenging activity of the sample or Trolox was plotted against its concentration to calculate IC_50_ (µg/mL). The samples were analyzed in three independent assays. In order to express the DPPH results as mmol TE/g extract, the IC_50_ (µg/mL) of Trolox was converted to mmol/mL using Trolox molecular weight (250.29 mg/mmol) and then dividing by the IC_50_ of each sample.

### 3.6. ORAC Antioxidant Activity

The ORAC (Oxygen Radical Absorbance Capacity) antioxidant activity was determined following a method previously described [47] using fluorescein as a fluorescence probe. The reaction was performed in 75 mM phosphate buffer (pH 7.4) at 37 °C. The final assay mixture consisted of AAPH (12 mM), fluorescein (70 nM), and either Trolox (1–8 µM) or the extract at different concentrations. Fluorescence was recorded every minute for 98 min in black 96-well untreated microplates (Nunc, Denmark), using a Polarstar Galaxy plate reader (BMG Labtechnologies GmbH, Offenburg, Germany) with 485-P excitation and 520-P emission filters. Fluostar Galaxy software version 4.11-0 (BMG Labtechnologies GmbH, Offenburg, Germany) was used to measure fluorescence. Fluorescein was diluted from a stock solution (1.17 mM) in 75 mM phosphate buffer (pH 7.4), while AAPH and Trolox solutions were freshly prepared. All reaction mixtures were prepared in duplicate and three independent runs were completed for each extract. Fluorescence measurements were normalized to the curve of the blank (no antioxidant). From the normalized curves, the area under the fluorescence decay curve (AUC) was calculated as:

$$AUC=1+\overset{i=98}{\underset{i=1}{\sum }}{\int^{\text{​}}}_{i}/{\int^{\text{​}}}_{0}$$

where *f*_0_ is the initial fluorescence reading at 0 min and *f_i_* is the fluorescence reading at time *i*. The net AUC corresponding to a sample was calculated as follows:

Net AUC = AUC_antioxidant_ − AUC_blank_

The regression equation between net AUC and antioxidant concentration was calculated. The ORAC value was estimated by dividing the slope of the latter equation by the slope of the Trolox line obtained for the same assay. Final ORAC values were expressed as mmol of Trolox equivalents (TE)/g of phenolic extract.

### 3.7. Statistical Analysis

In order to evaluate if the total phenolic contents (TPC) contributes to the antioxidant activity evaluated with DPPH and ORAC methodologies, a correlation analysis was carried out between TPC values with DPPH and ORAC results. Also, one-way analysis of variance (ANOVA) followed by Tukey’s post hoc test was applied to TPC, DPPH and ORAC values, and differences were considered significant at *p* < 0.05.

## 4. Conclusions

The qualitative analysis of phenolic-enriched extracts of the only commercial cultivars of *M. domestica* (Anna cultivar) and *P. domestica* (Satsuma cultivar) in Costa Rica, using UPLC-DAD-ESI-MS techniques, shows 52 compounds characterized, distributed as 21 proanthocyanidins, including procyanidin A-type and B-type dimers and trimers, B-type tetramer and pentamers, flavan-3-ol monomers and gallates; 15 flavonoids, including kaempferol, quercetin and naringenin derivatives, and eight phenolic acid derivatives in both fruits; as well as chalcones and isoprenoid glycosides in Anna apples. These findings constitute the first report of such a high number and diversity of compounds in skins of one single plum cultivar and of the presence of proanthocyanidin pentamers in apple skins. Also, it is the first time that such a large number of glycosylated flavonoids and proanthocyanidins are reported in skins and flesh of a single plum cultivar. Further, significant negative correlation was found for both apple and plum samples between TPC and DPPH antioxidant values, especially for plum fruits (*R* = −0.981, *p* < 0.05) as well as significant positive correlation between TPC and ORAC, also especially for plum fruits (*R* = 0.993, *p* < 0.05). DPPH and ORAC methods show high values for all samples, especially for fruits skins, thus indicating the potential value of these extracts. The presence of procyanidin tetramers and pentamers in apple skin could be responsible for the higher antioxidant potential, in agreement with reports indicating higher antioxidant values related to the presence of this type of procyanidin oligomers [20,21]. Further purification or fractioning of these extracts would be important to evaluate their structure-bioactivity relationship and, for instance, specific proanthocyanidin structures effect on epithelial gastrointestinal cancer cells, of particular relevance due to promising related results [3,48] and the fact that proanthocyanidins low absorption make gut epithelial cells [49] likely one of the main tissues where these compounds can actually exert their biological effects.

## Figures and Tables

**Compound No.**	**R**
3, 4	Galloyl
9, 15	H

**Compound No.**	**R**
36	H
24, 25, 31	(epi)catechin

**Compound No.**	**R**
1, 8, 13, 38, 42	H
14, 22, 23, 28, 30	(epi)catechin
26	(epi)catechin-(epi)catechin
27, 29	(epi)catechin-(epi)catechin-(epi)catechin

**Compound No.**	**O-R_1_**	**R_2_**	**R_3_**
18, 19	O-hexoside	H	H
32	H	H	hexoside
33, 34	O-pentosylhexoside	OH	H
37, 41	O-hexoside	OH	H
40	O-rutinoside	OH	H
43, 46	O-pentoside	OH	H
47	O-pentosylpentoside	OH	H
48, 49, 51	O-deoxyhexoside	OH	H
52	O-acetylhexoside	OH	H

**Compound No.**	**R_1_**	**R_2_**
5, 12	quinic acid	OH
6	hexoside	OH
7	H	H
10, 11	hexoside	H
17	quinic acid	H

**Compound No.**	**R_1_**	**R_2_**
35	OH	pentosylhexoside
39	OH	H
44, 45	H	Pentosylhexoside
50	H	H

**Compound No.**	**R**
20, 21	pentosylhexoside

**Table 1:** Extraction yield and total phenolic content.

Sample	Lyophilization Yield (%) ^1^	Extraction Yield (%) ^2^	Total Phenolic Content (mg GAE/g Extract) ^3,4^
*M. domestica*			
Anna-Skin	11.6	1.20	619.6 ^a,b^ ± 19.5
Anna-Flesh	13.8	0.51	576.0 ^a^ ± 20.9
*P. domestica*			
Satsuma-Skin	13.1	2.74	640.3 ^b^ ± 22.7
Satsuma-Flesh	17.1	1.24	515.2 ^c^ ± 17.3

^1^ g of dry material/g of fresh weight expressed as %. ^2^ g of extract/g of dry material expressed as %. ^3^ Values are expressed as mean ± Standard Deviation (S.D.). ^4^ Different superscript letters in the column indicate differences are significant at *p* < 0.05 using one-way analysis of variance (ANOVA) with a Tukey post hoc as statistical test. GAE: gallic acid equivalents.

**Table 2:** Profile of phenolic compounds identified by UPLC-DAD-ESI-TQ-MS analysis for apple and plum samples.

No.	Tentative Identification	t_R_ (min)	λ_max_ (nm)	[M − H]^−^	Formula	MS^2^ Fragments (% Abundance)	Apple Anna Skin	Apple Anna Flesh	Plum Satsuma Skin	Plum Satsuma Flesh
Proanthocyanidins										
1	Procyanidin B-type dimer	2.69	277	577.1344	C_30_H_26_O_12_	[577]: 289(28), 407(79), 425(100), 451(49), 559(66)	x			
3	(epi)catechin 3-O-gallate	3.96	284	441.0818	C_22_H_18_O_10_	[441]: 153(31), 289(35), 315(100)	x		x	x
4	(epi)catechin 3-O-gallate	5.40	284	441.0819	C_22_H_18_O_10_	[441]: 153(32), 289(28), 315(100)				x
8	Procyanidin B-type dimer	8.44	279	577.1344	C_30_H_26_O_12_	[577]: 289(50), 407(70), 425(100), 451(80), 559(42)	x	x		
9	Catechin	8.87	289	289.0709	C_15_H_20_O_6_	[289]: 205(38),245(100)	x		x	
13	Procyanidin B-type dimer	12.75	282	577.1344	C_30_H_26_O_12_	[577]: 289(35), 407(57), 425(100), 451(56), 559(28)	x	x	x	x
14	Procyanidin B-type trimer	13.20	282	865.1956	C_45_H_38_O_18_	[865]: 577(43), 695(100), 713(39), 739(60)				x
15	Epicatechin	13.97	280	289.0707	C_15_H_14_O_6_	[289]: 205(35), 245(100)	x	x	x	x
22	Procyanidin B-type trimer	17.88	279	865.1956	C_45_H_38_O_18_	[865]: 577(54), 695(100), 713(37), 739(71)	x		x	
23	Procyanidin B-type trimer	18.16	278	865.1956	C_45_H_38_O_18_	[865]: 577(61), 695(100), 713(33), 739(67)		x		x
24	Procyanidin A-type trimer	18.98	278	863.1798	C_45_H_36_O_18_	[863]: 575(100), 711(63)			x	x
25	Procyanidin A-type trimer	19.37	277, 517	863.1798	C_45_H_36_O_18_	[863]: 575(100), 711(63)				x
26	Procyanidin B-type tetramer	19.70	279, 517	1153.2603	C_60_H_50_O_24_	[1153]: 575(43), 577(46), 863(62), 865(100), 983(87), 1001(37), 1027(66)	x	x		x
27	Procyanidin B-type pentamer	20.54	280	1441.3229	C_75_H_62_O_30_	[1441]: 1315(43), 1151(70), 863(68), 635(100), 577(40)	x			
28	Procyanidin B-type trimer	21.23	279	865.1956	C_45_H_38_O_18_	[865]: 407(45), 577(59), 695(100), 713(66), 739(73)		x		x
29	Procyanidin B-type pentamer	21.83	278	1441.3229	C_75_H_62_O_30_	[1441]: 1315(33), 1151(69), 863(95), 635(100), 577(60)	x			
30	Procyanidin B-type trimer	22.21	279	865.1956	C_45_H_38_O_18_	[865]: 575(46), 577(53), 695(100), 713(44), 739(84)		x		
31	Procyanidin A-type trimer	23.54	282	863.1798	C_45_H_36_O_18_	[863]: 575(100), 711(58)				x
36	Procyanidin A-type dimer	25.19	279	575.1185	C_30_H_24_O_12_	[575]: 289(35), 449(100)		x	x	x
38	Procyanidin B-type dimer	27.21	276	577.1344	C_30_H_26_O_12_	[577]: 289(34), 407(60), 425(100), 451(78), 559(31),				x
42	Procyanidin B-type dimer	28.62	279	577.1344	C_30_H_26_O_12_	[577]: 289(50), 407(65), 425(100), 451(76), 559(44)		x		
Glycosylated flavonols										
18	Kaempferol-hexoside	14.97	280, 351	447.0922	C_21_H_20_O_11_	[447]: 284(70), 285(100)	x			
19	Kaempferol-hexoside	15.90	278, 360, 516	447.0922	C_21_H_20_O_11_	[447]: 284(23), 285(100)				x
32	Naringenin-hexoside	23.97	278, 351	433.1131	C_21_H_22_O_10_	[433]: 271(100)	x			
33	Quercetin-pentosylhexoside	24.12	279, 351	595.1284	C_26_H_28_O_16_	[595]: 300(100), 301(41)	x			
34	Quercetin-pentosylhexoside	24.72	281, 357	595.1284	C_26_H_28_O_16_	[595]: 300(100), 301(40)			x	
37	Quercetin-hexoside	26.57	255, 350	463.0875	C_21_H_20_O_12_	[463]: 300(36), 301(100)	x			
40	Quercetin-rutinoside	27.60	255, 360	609.1440	C_27_H_30_O_16_	[609]: 300(31), 301(100)	x	x	x	x
41	Quercetin-hexoside	27.79	252, 351	463.0875	C_21_H_20_O_12_	[463]: 300(28), 301(100)		x	x	x
43	Quercetin-pentoside	29.28	258, 355	433.0769	C_20_H_18_O_11_	[433]: 300(29), 301(100)	x	x	x	
46	Quercetin-pentoside	30.69	258, 347	433.0769	C_20_H_18_O_11_	[433]: 301(100)			x	
47	Quercetin-pentosylpentoside	31.21	258, 354	565.1184	C_25_H_26_O_15_	[565]: 300(100), 301(16)			x	
48	Quercetin-deoxyhexoside	32.46	355	447.0922	C_21_H_20_O_11_	[447]: 300(26), 301(100)			x	
49	Quercetin-deoxyhexoside	32.57	284	447.0922	C_21_H_20_O_11_	[447]: 300(30), 301(100)		x		
51	Quercetin-deoxyhexoside	33.27	281	447.0922	C_21_H_20_O_11_	[447]: 300(30), 301(100)	x			
52	Quercetin-acetylhexoside	33.51	354	505.0975	C_23_H_22_O_13_	[505]: 300(63), 301(100)			x	
Acids and derivates										
2	Protocatechuic acid	3.36	280	153.0191	C_7_H_6_O_4_	[153]: 109(100)	x		x	
5	Caffeoylquinic acid isomer	5.95	323	353.0869	C_16_H_18_O_9_	[353]: 191(100), 179(71)			x	x
6	Caffeoyl hexoside	7.23	331	341.0872	C_15_H_18_O_9_	[341]: 161(37), 179(100)			x	x
7	Coumaric acid	8.30	313	163.0398	C_9_H_6_O_3_	[163]: 119(100)			x	
10	*p*-coumaroyl-hexoside	9.94	297	325.0921	C_15_H_18_O_8_	[325]: 145(100), 163(92), 187(49)	x			x
11	*p*-coumaroyl-hexoside	10.27	314	325.0921	C_15_H_18_O_8_	[325]: 145(100), 163(87), 187(50)		x	x	x
12	Caffeoylquinic acid isomer	11.10	270, 313	353.0869	C_16_H_19_O_9_	[353]: 191(100), 145(46)	x	x		
16	Shikimic acid	14.44	316	173.0454	C_7_H_10_O_5_	[173]: 93(100),111(43)			x	
17	*p*-coumaroylquinic acid	14.49	311	337.0927	C_16_H_18_O_8_	[337]: 173(100)		x		
Chalcones										
35	3-hydroxyphloretin-pentosylhexoside	24.87	281	583.1660	C_26_H_32_O_15_	[583]: 289(100)	x	x		
39	3-hydroxyphloretin	27.29	283	289.0716	C_15_H_14_O_6_	[289]: 167(100), 245(49), 271(81)		x		
44	Phloretin-pentosilhexoside	30.11	284	567.1704	C_26_H_32_O_14_	[567]: 273(100)	x	x		
45	Phloretin-pentosilhexoside	31.04	283	567.1704	C_26_H_32_O_14_	[567]: 273(100)		x		
50	Phloretin	32.78	283	273.0767	C_15_H_14_O_5_	[273]: 167(100)	x	x		
Other compounds										
20	Vomifoliol-pentosilhexoside	16.80	281	517.2280	C_24_H_38_O_12_	[517]: 205(100), 385(58)	x	x		
21	Vomifoliol-pentosilhexoside	17.10	281	517.2280	C_24_H_38_O_12_	[517]: 205(100), 385(64)	x	x		

**Table 3:** DPPH and ORAC antioxidant activity.

Sample	DPPH ^1,2^		ORAC ^1,2^
	IC_50_ (μg/mL)	(mmol TE/g Extract)	(mmol TE/g Extract)
*M. domestica*			
Anna-Skin	4.54 ^a^ ± 0.06	1.24 ^a^ ± 0.02	16.78 ^a^ ± 0.25
Anna-Flesh	6.64 ^b^ ± 0.12	0.85 ^b^ ± 0.01	11.22 ^b^ ± 0.13
*P. domestica*			
Satsuma-Skin	5.19 ^c^ ± 0.12	1.08 ^c^ ± 0.02	14.55 ^c^ ± 0.21
Satsuma-Flesh	5.95 ^d^ ± 0.14	0.94 ^d^ ± 0.03	13.02 ^d^ ± 0.29

^1^ Values are expressed as mean ± S.D. ^2^ Different superscript letters in the same column indicate differences are significant at *p* < 0.05 using ANOVA with a Tukey post hoc as statistical test. ORAC: oxygen radical absorbance capacity; DPPH: 2,2-diphenyl-1-picrylhidrazyl method.
